# Forensic genetics

**DOI:** 10.1080/20961790.2018.1489445

**Published:** 2018-07-18

**Authors:** Chengtao Li

**Affiliations:** Shanghai Key Laboratory of Forensic Medicine, Shanghai Forensic Service Platform, Academy of Forensic Sciences, Shanghai, China

Like other subjects, forensic genetics gradually emerged and developed through long-term social practice. Following the discovery of the ABO blood groups by Landsteiner in 1900 [1], human blood type was used in identification, and forensic genetics entered the scientific age. In 1910, the French criminologist Edmond Locard proposed the Locard’s exchange principle [2] and stated that “every contact leaves a trace,” which laid the foundation for modern forensic science. In 1926, Thomas Hunt Morgan [3] proposed a theory of genes, which provided the basis for the development of forensic genetics. In 1953, the discovery of the double-helical structure of DNA enabled the start of forensic genetics research at the molecular level [3].

One of the central aspects of forensic genetics is the use of genetic markers, which are the easily identifiable phenotypes of genotypes. Genetic markers generally have features such as strong polymorphisms, codominant expression and ease of observation and recording. With the development of genetics, the use of genetic markers has also developed steadily. To date, the development of genetic markers has gone through four major stages characterized by the use of morphological markers, cytological markers, biochemical markers and molecular markers. In particular, this present special issue provides an introduction to the application and development for molecular markers in forensic areas.

During the early 1990s, short tandem repeats (STRs) were used for the first time as key molecular markers in human paternity testing. The first forensic multiplex amplification STR kit was developed by Britain’s Forensic Science Service (FSS) and included four genetic loci: *TH01, vWA, FES/FPS* and *F13A1*. Today, commercial STR kits can generally detect 15–20 STR loci at one time. With the advancement of fluorescent marker techniques, six-color fluorescent marker STR kits have appeared on the market, and 25–30 STR loci can be detected. The International Society for Forensic Genetics (ISFG) has drafted a guide to the forensic validation of STR kits, and this guide provides excellent standards and guidance for the forensic application of STR kits.

The rapid development of massively parallel sequencing (MPS) has attracted widespread attention among forensic genetics researchers. STR typing based on capillary electrophoresis can only reflect differences in length; however, MPS can also reveal differences in the internal sequence and flanking structure of STRs, which tremendously increases the available genetic information and provides a new possible method for difficult cases involving complex kinship identification and the resolution of mixtures. In addition, the development of MPS has also promoted metagenomics research. In recent years, metagenomics has developed as a new branch of microbial ecology that addresses the microbial composition and diversity of different environments, and microbial community members’ relationships with each other and their environment. Metagenomics is gradually being applied in areas connected with forensic identification such as individual identification, identification of the source of biological stains in crime scenes and the detection of drug abuse, which has also brought new research opportunities.

Since forensic polymorphic STR loci are limited in number and cannot provide more functional information, which hampers the effectiveness of the application in forensic community, a new type of genetic marker, single nucleotide polymorphisms (SNPs), may replace STRs in the future, although this process may take a considerable period of time. Compared with STR loci, SNP sites have a lower mutation rate, approximately 10^−8^ [4], and the amplification products of individual SNP sites can be very short, which makes SNPs suitable for the analysis of highly degraded forensic samples. SNP analysis already plays an important role in the identification of individuals after mass disasters. Furthermore, SNPs are linked with multiple phenotypes, such as individual’s skin color, sclera color, hair curliness, information concerning ethnic origin, differences in alcohol metabolism and susceptibility to hereditary diseases. The aforementioned advantages of SNPs have all been successfully applied, and SNP analysis occupies an unsurpassed position in forensic practice. Thanks to ongoing research advances, reliance on SNP testing to obtain DNA portraits no longer appears to be an impossible dream.

Apart from DNA-level genetic markers, RNA-level genetic markers have also become a new type of marker and research hotspot in forensic genetics. In 2000, progress in RNA research was acclaimed by *Science* as a major scientific breakthrough. A series of groundbreaking advances in RNA research during the past 20 years have enabled RNA to escape from the brilliance of DNA’s aura, have changed RNA from the “supporting role” to the “leading role” and have made RNA a new challenge to DNA’s central position. In the field of forensics, steady progress is being made in applied research involving the use of RNA to identify racial affiliation, the identification of bodily fluids, the determination of time of death and the analysis of injuries. In addition, thanks to their advantages of convenience, economy, speed and precision, protein-level genetic markers offer great promise in forensic genetics and related fields.

In summary, the application of genetic markers has advanced in pace with the scientific knowledge and technological capabilities, which has expanded and changed the scope of forensic genetics research. From the dual perspectives of the development of genetic markers and their applications, the papers in this special issue explain that forensic genetics research is no longer limited to DNA-level markers but also includes RNA-level and protein-level markers. The scope of forensic genetics research is no longer limited to conventional determination of kinship and individual identification; it has steadily branched out into areas such as forensic clinical medicine, forensic pathology and forensic psychiatry, yielding many new findings and applications.
